# Current landscape of exosomes in tuberculosis development, diagnosis, and treatment applications

**DOI:** 10.3389/fimmu.2024.1401867

**Published:** 2024-05-23

**Authors:** Xuezhi Sun, Wei Li, Li Zhao, Ke Fan, Fenfen Qin, Liwen Shi, Feng Gao, Chunlan Zheng

**Affiliations:** ^1^ Department of Tuberculosis III, Wuhan Pulmonary Hospital, Wuhan, Hubei, China; ^2^ Department of Endocrinology, Union Hospital, Tongji Medical College, Huazhong University of Science and Technology, Wuhan, Hubei, China

**Keywords:** tuberculosis, extracellular vesicles, exosome, immune regulation, diagnosis, therapeutic applications

## Abstract

Tuberculosis (TB), caused by the bacterial pathogen *Mycobacterium tuberculosis (MTB)*, remains one of the most prevalent and deadly infectious diseases worldwide. Currently, there are complex interactions between host cells and pathogens in TB. The onset, progression, and regression of TB are correlated not only with the virulence of *MTB* but also with the immunity of TB patients. Exosomes are cell-secreted membrane-bound nanovesicles with lipid bilayers that contain a variety of biomolecules, such as metabolites, lipids, proteins, and nucleic acids. Exosome-mediated cell−cell communication and interactions with the microenvironment represent crucial mechanisms through which exosomes exert their functional effects. Exosomes harbor a wide range of regulatory roles in physiological and pathological conditions, including *MTB* infection. Exosomes can regulate the immune response, metabolism, and cellular death to remodel the progression of *MTB* infection. During *MTB* infection, exosomes display distinctive profiles and quantities that may act as diagnostic biomarkers, suggesting that exosomes provide a revealing glimpse into the evolving landscape of *MTB* infections. Furthermore, exosomes derived from *MTB* and mesenchymal stem cells can be harnessed as vaccine platforms and drug delivery vehicles for the precise targeting and treatment of TB. In this review, we highlight the functions and mechanisms through which exosomes influence the progression of TB. Additionally, we unravel the critical significance of exosomal constituents in the diagnosis and therapeutic applications of TB, aiming to offer novel perspectives and strategies for combating TB.

## 1 Introduction

Tuberculosis (TB) is one of the oldest known diseases and one of the most lethal public threats in the world (1). Unlike other common infectious diseases, *Mycobacterium tuberculosis (MTB)*, the causative bacteria of TB, can protect itself from removal by the immune system through complex interactions with the host immune system (2). This complex interaction contributes to the treatment difficulty and widespread prevalence of TB. The activating and coordinating role of innate and adaptive immunity during *MTB* infection possesses a decisive role in both clearance and the inability to control *MTB*.

Exosomes are lipid bilayer vesicle structures that can be loaded with a variety of biologically viable molecules responsible for adjacent or distant cellular communication and regulation (3). In TB, *MTB*-infected host cells exhibit active exosome synthesis and secretion. Surprisingly, *MTB* is also capable of synthesizing and releasing vesicles despite its sophisticated cell wall structure (4). Vesicles from *MTB* are comparable in size (20–300 nm) to cell-secreted vesicles, with abundant lipid and protein compositions. Numerous proteins involved in host−-pathogen interaction processes, as well as a number of nucleic acids, have been found in these vesicles (5, 6). Both *MTB*-secreted vesicles (MEVs) and exosomes secreted by infected cells play significant roles in TB progression (7). For instance, Prados-Rosales et al. reported that an iron-deficient environment enhances *MTB* secretion of microvesicles containing mycobactin, in which mycobactin can assist iron-restricted *MTB* in acquiring iron and promote their replication (8). The type and abundance of exosomal components and contents change as TB progresses, which is an important basis for their use as diagnostic and therapeutic markers.

Thus, exosomes are important information carriers for interactions between TB and the host. Exosomes modify the course of TB by regulating signaling molecules, inflammatory intensity, immune activation, and escape. In this review, we emphasize the roles and mechanisms of exosomes in remodeling the TB progression. We also decipher the pivotal role of exosomal components in the diagnosis and therapeutic potential of TB, hoping to provide novel perspectives and strategies for combating TB.

## 2 TB and immune regulation

*MTB* infection persists as a global health challenge, precipitating millions of new cases annually with an approximate mortality toll of 1.5 million. The pathogen predominantly afflicts the pulmonary system but can also instigate disseminated disease, affecting multiple organs (9). TB continues to pose a serious threat to the health of people around the world, especially in less developed regions. Current diagnostic methods are either complicated or time-consuming. The standard treatment regimen for TB requires treatment periods of up to six to nine months, which can easily impair patient adherence to treatment, thereby compromising treatment efficacy and leading to the emergence of drug-resistant strains (10). The efficiency of TB diagnosis and treatment is also unsatisfactory, although updated guidelines are provided annually to guide TB diagnosis and treatment (11).

**Figure 1:**
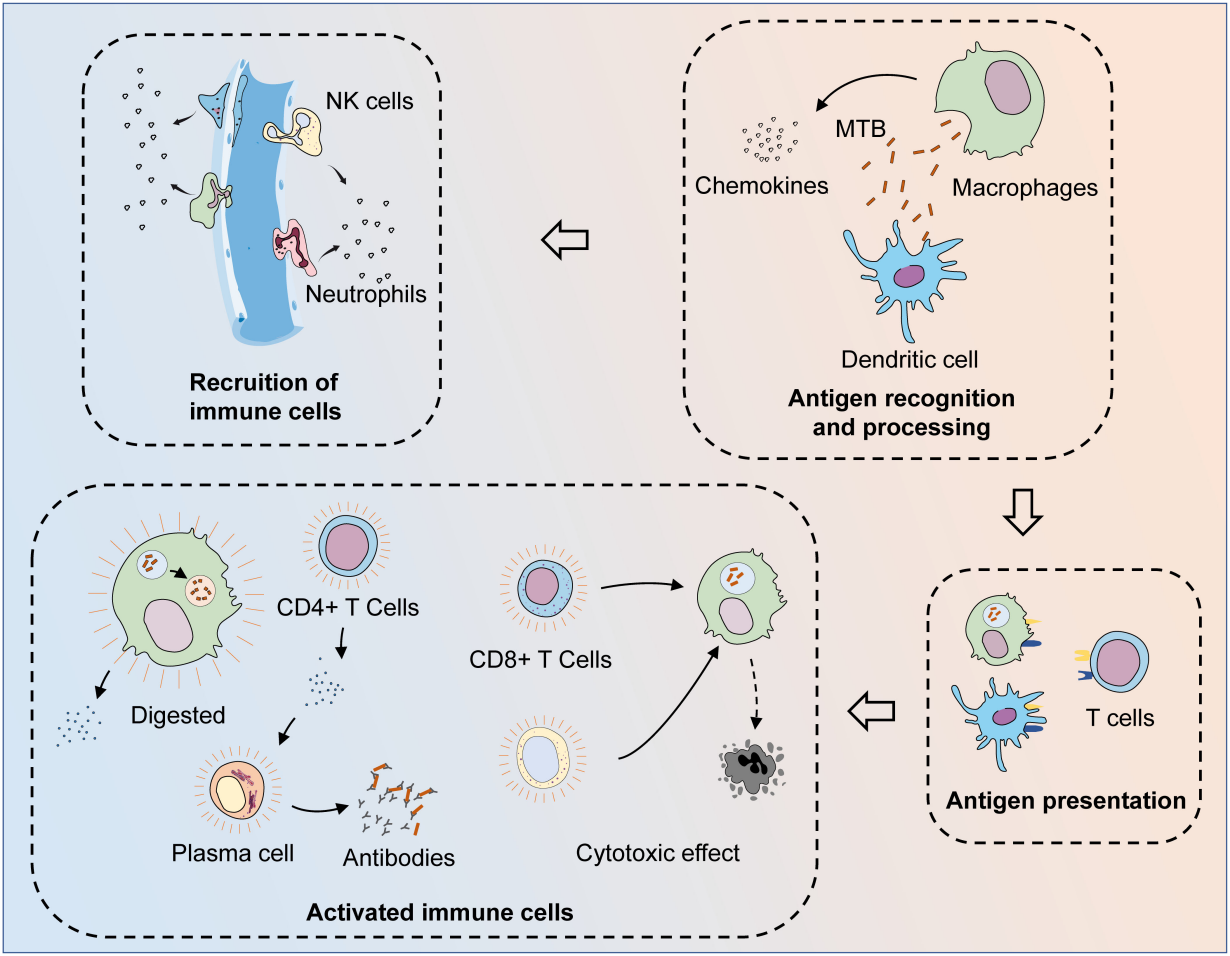
The process of the immune system fighting *MTB* infection. After invading the body, *MTB* are first recognized and phagocytosed by antigen-presenting cells (APCs), such as macrophages and dendritic cells. At the same time, macrophages could also phagocytose *MTB* and digest them by facilitating phagosome maturation, while *MTB* can resist the process of being cleared by interacting with the host immune system. After *MTB* is phagocytosed, APCs can release chemokines to recruit other immune cells to the infection site. After the phagocytosed *MTB* are processed, their specific antigens are exposed and presented to T cells, thus activating adaptive immunity. Through the synthesis and secretion of specific antibodies and cytotoxic effects, adaptive and innate immunity together exert anti-TB effects. Various cytokines are released during the activated immune process, while macrophages that fail to clear the intracellular *MTB* successfully are also lysed, leading to the clearance of the *MTB* present within them.

*MTB* is recognized and phagocytosed by macrophages and dendritic cells (DCs) upon first entering the body (12, 13). These antigen-presenting cells can process phagocytosed bacteria and then activate adaptive immunity through antigen presentation, leading to a synergistic anti-TB effect. Both activated CD8+ T cells and activated natural killer (NK)/NKT cells can lyse infected cells and limit *MTB* infection (14, 15). Humoral immunity is also involved in the defense process against *MTB* infection (16). In the other aspect, macrophages are the main players against *MTB*. After the formation of phagosomes by engulfing *MTB*, macrophages can degrade *MTB* by promoting phagosomal acidification and fusion with lysosomes (17). However, *MTB* can resist the fate of being eliminated by interacting with the immune system, for example, by utilizing macrophages as a shelter and surviving in the host (18). This means that a complete cure for TB is very complicated and difficult. *MTB* infection can result in both active tuberculosis (ATB) and latent tuberculosis (LTB) states, while LTB can activate again in the presence of a compromised host immune system and turn into ATB. In the context of the progressive increase in the incidence of various chronic diseases, TB may be one of the main causes of their prevalence and spread (19, 20) (**Figure 1**).

## 3 Exosome characterization and formation

Exosomes are defined as a category of extracellular vesicles (EVs) in the size range of 30 to 150 nm, possessing a phospholipid bilayer. The surface of exosomes contains abundant conserved proteins, such as tetratransmembrane proteins (CD9, CD63, and CD81), endosomal-sorting complex required for transport (ESCRT) proteins (Alix and TSG101), heat shock proteins (Hsp60, Hsp70, and Hsp90), cell-specific antigen-presenting molecules, glycoproteins, and some adhesion molecules (21, 22). Exosomes are secreted by almost all types of cells and are ubiquitous in a wide range of body fluids with spatially and temporally specific expression. Exosomes perform a wide range of regulatory roles in physiological and pathological conditions, including innate and adaptive immunity, cellular activity, and programmed cell death (PCD) (23).

The cargoes inside exosomes, including proteins, metabolites, lipids, DNA, and non-coding RNAs (ncRNAs), reflect the molecular processing inside parent cells. Among them, miRNAs are one of the most abundant RNA species within exosomes and are also the most studied type of ncRNAs (24–26). Upon contact with target cells, exosomes can enter the intracellular compartment via receptor-ligand interactions, direct membrane fusion, and endocytosis/phagocytosis (27). The binding of transmembrane ligands on the surface of exosomes to receptors on recipient cells can activate downstream cascade signaling (28). On the other hand, exosomes can also bind directly to the plasma membrane and release their contents into target cells, thus exerting cellular regulation (29).

**Figure 2:**
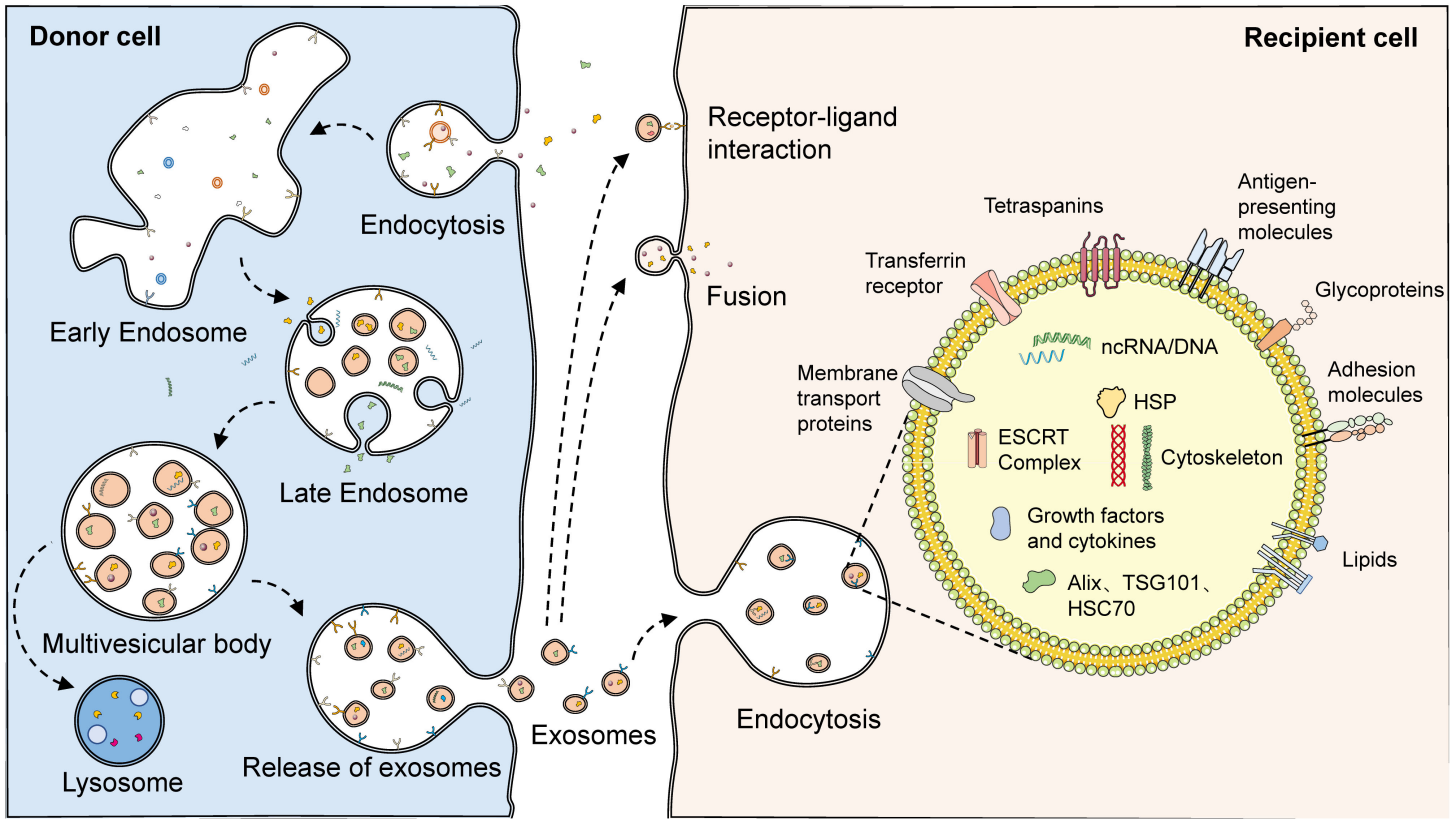
The biogenesis and structure of exosomes. With endocytosis, cells can ingest extracellular macromolecules and vesicles, forming early endosomes that encapsulate these cargoes. In some cases, early endosomes can also fuse with each other. Cargoes not involved in recirculation finally become part of the late endosome with the maturation of the early endosome. During the maturation of late endosomes, variable proteins, lipids, and nucleic acids are encapsulated through complex sorting pathways. With the membrane of the late endosome budding inward, intraluminal vesicles encasing the cargoes are formed, which consequently develop multivesicular endosomes. Multivesicular endosomes have two fates: fusing with lysosomes, which then results in the degradation of the cargoes in intraluminal vesicles, or fusing with the plasma membrane, which then releases the luminal vesicles as exosomes. A variety of proteins and lipids, such as membrane transport proteins, transferrin receptors, tetraspanins, antigen-presenting molecules, glycoproteins, and adhesion molecules, existed on the surface of exosomes. However, abundant proteins, lipids, and nucleic acids are also present in their interior. Released exosomes can complete communication with receptor cells through three pathways: receptor-ligand interaction, fusion, and endocytosis.

The biogenesis of exosomes can be divided into three main phases. First, the plasma membrane forms early endosomes by budding inward. Recognition and endocytosis of molecular cargoes initiates the exosome biosynthetic pathway. The plasma membrane forms cup-shaped structures by inward budding, and these vesicles containing cell surface proteins and external cargoes form early endosomes by further fusion (30). In some cases, early endosomes can also fuse with each other (3). The formation of early endosomes mediates the primary sorting and homing of endocytosed carriers, which are recirculated, fused to lysosomes, and released extracellularly after degradation and exosome formation. Second, early endosomes are converted to late endosomes to form multivesicular bodies (MVBs) containing luminal vesicles. Cargoes that are not recirculated participate in the maturation of early sorting endosomes to form late sorting endosomes. Protein sorting can proceed by ESCRT-dependent or non-ESCRT mechanisms (31–33). After sorting, the membranes of late-sorted endosomes can sprout again toward the interior of the endosome, forming intraluminal vesicles (ILVs) that encapsulate the cargo, which in turn form multivesicular endosomal structures or MVBs (34, 35). Finally, the fusion of late MVBs to the plasma membrane results in the release of internal ILVs as exosomes, which are degraded if these MVBs are fused to lysosomes. When soluble TB proteins are exogenously added to RAW264.7 or human HEK293 cells, these proteins are endocytosed, ubiquitinated, and released via exosomes (36). Ubiquitination is a sufficient modification for the translocation of soluble proteins from the phagocytic/endocytotic network to exosomes. Various regulatory proteins, such as the RAB family, VAMP3, SNARE, and others, are also involved in exosome release (37–39) (**Figure 2**).

## 4 The roles and mechanisms of exosomes in TB

### 4.1 Immune defense

Exosomes are expected to serve as key vehicles for *MTB* pathogen-associated molecular patterns (PAMPs), through which *MTB*s inhibit host immune responses, and promote immune escape and survival. The dynamic change and balancing tendency of cytokines is a manifestation of immune effect in many immune-related diseases (40–43). *MTB* conjugated to toll-like receptor 2 (TLR2) triggers mast cells to release exosomes containing high levels of chemokine (C-C motif) ligand 2 (CCL2), IL-4, and IL-13, and causes macrophage M2 polarization, which potentiates the immune escape effect of *MTB* (44). Singh et al. demonstrated that exosomes released from *MTB*-infected cells restrained IFN-γ-mediated activation of naïve macrophages (45). In their further study, exosomes released from *MTB*-infected RAW264.7 cells promoted the secretion of chemokines and induced macrophage and splenocyte migration (46). In addition, serum exosomes from infected mice effectively promoted recruitment to macrophages *in vivo*, while intranasal exosomes also recruited CD11b+ cells into the lungs. During *MTB* infection, exosomes are important dissemination vectors for host cell recruitment and colonization.

*MTB*-infected macrophages and DCs possess limited capability to present antigens to CD4+ T cells, but the T-cell response is robust, suggesting alternative T-cell activation processes. Antigen presentation is fundamental for the activation of *MTB*-resistant T cells. Cellular components, such as apoptotic bodies, necrotic debris, and exosomes, might be important origins of antigens. Smith et al. demonstrated that exosomes were an important source of antigens, partially contributing to T cell function activity during *MTB* infection (47). Ramachandra et al. confirmed that exosomes derived from *MTB*-infected macrophages, harbored *MTB* peptide-MHC-II complexes, which further processed antigen presentation and activated antimicrobial T-cell responses (48). The PE_PGRS protein encoded by *MTB* Rv1818c could affect bacterial cell structure and induce distinct B-cell responses (49). Balaji et al. found that exosomal Rv1818c could be released by *MTB*-infected BM-DCs and macrophages, and thus triggered Jurkat T-cell apoptosis (50).

HIV and TB are known to be susceptible to co-infection, play complementary roles in host co-infection, and contribute to the global disease burden. Tyagi et al. proved that exosomes from *MTB*-infected macrophages were complex carriers containing HIF-1α, galectins, and HSP90, which mediated oxidative stress, inflammation, and consequent HIV-1 reactivation (51). This study emphasized the pivotal role of redox and energy metabolism as important processes influencing HIV-TB synergy.

### 4.2 Pro-inflammation

Exosomes containing PAMPs are important for initiating proinflammatory responses against *MTB*. Exosomes released from *MTB*-infected macrophages initiate proinflammatory responses via TLRs and myeloid differentiation factor 88 (MyD88) both *in vitro* and *in vivo* (52). Bhatnagar et al. found that macrophages infected with *Mycobacterium avium* could release exosomes containing glycopeptide lipids (GPL) (53). Through pathways dependent on TLR2, TLR4, and MyD88, these exosomes could perform immunosurveillance functions by stimulating pro-inflammatory responses in receptor macrophages. Singh et al. showed that exosomes released from *MTB*-infected macrophages elicited the differentiation of naïve monocytes, and produced active macrophages through MAPK-dependent signaling activated by MK-2 and NF-κβ (54). The release of these exosomes was driven by AKT phosphorylation and was associated with Rab7a and Rab11a. Although no direct evidence has been observed that exosomes secreted by infected macrophages are involved in the pro-inflammatory process in TB infection, available studies indicated that cells infected by M. avium could release exosomes containing components of bacterial origin. While resting macrophages were treated with these exosomes individually, it could be found that the expression of CD80 and CD86 and the secretion of TNF-α and IFN-γ in macrophages were enhanced in the macrophages, similar as those of M. avium-infected macrophages (55). Proteomic analysis demonstrated that, compared to those in exosomes from uninfected macrophages, two actin isoforms, guanine nucleotide-binding protein β-1, cofilin-1 and peptidyl-prolyl cis-trans isomerase A, were differentially expressed in exosomes secreted by infected macrophages. Also, exosomes secreted from infected MSCs could induce pro-inflammatory responses *in vivo* and *in vitro* conditions. Therefore, we hypothesized that exosomes from infected macrophages were also involved in the pro-inflammatory process in TB infection.

### 4.3 Metabolism

Certain *MTB* strains regulate the metabolic reprogramming of the host during infection to ensure a continuous supply of the necessary nutrients to modulate the immune response and survive long term *in vivo* (56). Alipoor et al. estimated the miRNA signature of exosomes released from human monocyte-derived macrophages (MDMs) infected with the *Mycobacterium bovis* bacillus Calmette-Guérin (BCG) vaccine (57). BCG-infected MDMs could release exosomal miR-1224, miR-1293, miR-425, and other miRNAs related to metabolism and energy production, suggesting possible metabolic changes after *MTB* infection. Wu et al. analyzed the proteomics of plasma exosomes in drug-resistant TB (DR-TB) and reported 16 up-regulated proteins and 10 down-regulated proteins in DR-TB patients compared with non-DR-TB patients (58). The apolipoprotein family, the major down-regulated proteins, including APOA1, APOB, and APOC1, might regulate cholesterol metabolism through exosome-mediated transport functions and represent an important pathogenetic mechanism for DR-TB.

### 4.4 Apoptosis and autophagy

Apoptosis is a genetically controlled PCD mode that sequentially and efficiently removes damaged cells, such as those resulting from DNA damage, and can be triggered by intrinsic or extrinsic pathways (59). Zhang et al. presented that the expression of miR-20b-5p was significantly reduced in both *MTB*-infected macrophages and their exosomes (60). Up-regulation of miR-20b-5p inhibited cell viability and induced apoptosis in macrophages by targeting MCL-1, thus demonstrating the feasibility and efficacy of miR-20b-5p and exosomes for treating TB.

**Figure 3:**
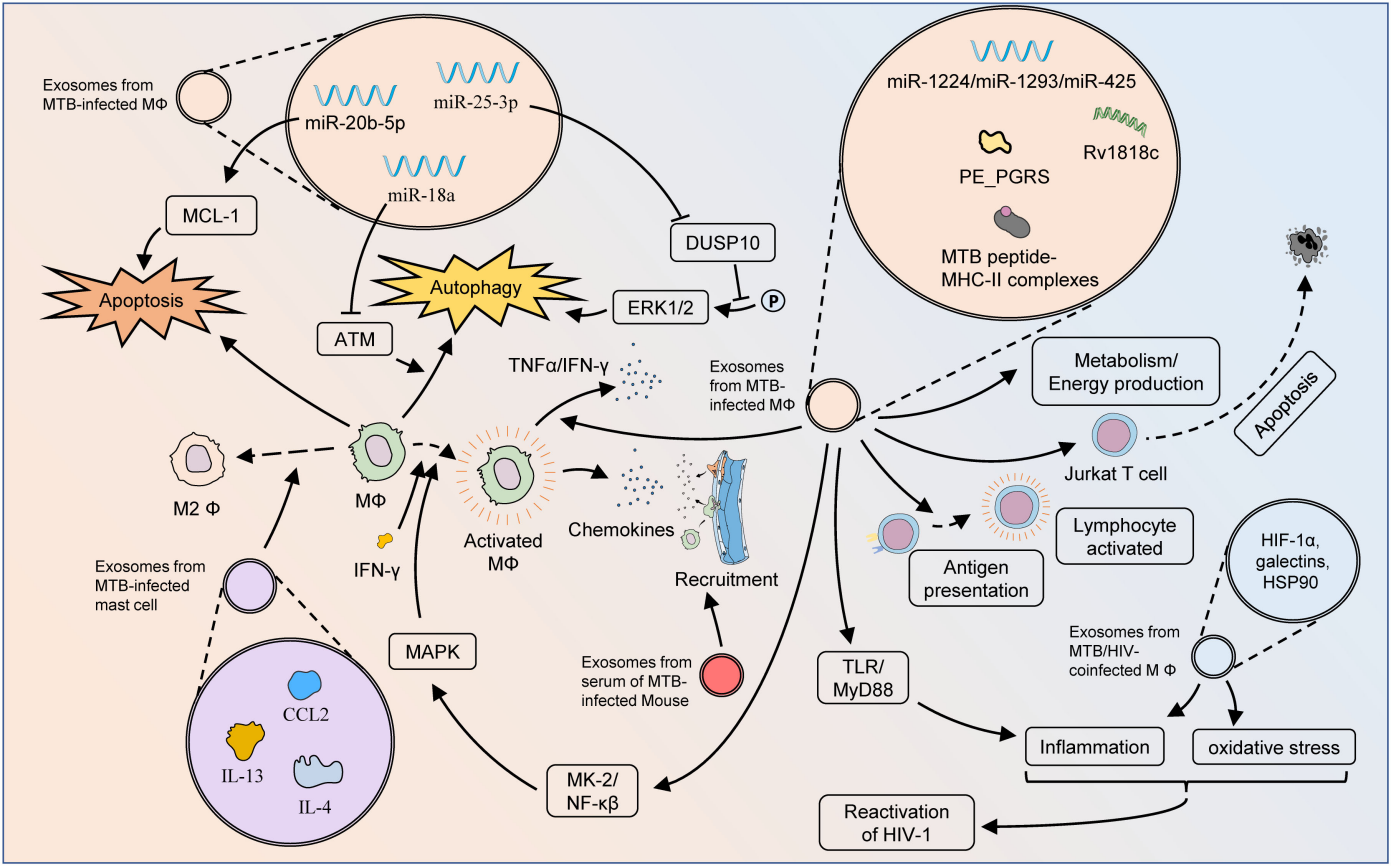
Mechanisms of exosomes influencing pathologic processes of TB. Exosomes from *MTB*-infected macrophages can affect cellular bioactivity through multiple pathways. The *MTB* peptide-MHC-II complexes present therein are involved in antigen presentation and activation of adaptive immunity, but exosomes from *MTB*-infected macrophages have also been found to induce apoptosis in Jurkat T cells. The autophagy and apoptosis of macrophages might also be regulated by exosomal miRNAs. Via TLR/MyD88, exosomes from *MTB*-infected macrophages can initiate a pro-inflammatory response to fight *MTB* infection. Moreover, these exosomes could activate macrophages through the MK-2-NF-κβ-MAPK pathway, and also stimulate the M1 polarization of macrophages as well as the secretion of TNF-α and IFN-γ. Exosomes from *MTB*-infected macrophages play an important role in metabolic pathways, which greatly influence the pathogenesis of DR-TB. Exosomes from *MTB*-infected mast cells contain substantial amounts of CCL2, IL-4, and IL-13, which lead to M2 polarization of macrophages and exacerbation of *MTB* infection. Exosomes from the serum of *MTB*-infected mice could enhance macrophage recruitment. Exosomes from the serum of HIV-TB co-infected patients could promote the inflammatory response and oxidative stress *in vivo*, thereby facilitating the reactivation of HIV.

Autophagy, another form of PCD, is a highly conserved lysosomal process utilized by eukaryotes to degrade cytoplasmic proteins and damaged organelles as an intracellular self-repair mechanism (61). Host cells, such as macrophages, can degrade pathogens through elaborate autophagy induction after *MTB* infection, while *MTB* can also persist in replication by inhibiting phagosome maturation and evading immune killing (62). Enhanced autophagy improves antimicrobial defense against *MTB* infection. Autophagy-based activation of gene therapy and drug therapy is a promising strategy for use in the treatment of *MTB* infections, even drug-resistant strains. Yuan et al. confirmed that *MTB*-infected RAW264.7 cells and their secreted exosomes both induced increased expression of miR-18a (63). Mechanistically, miR-18a down-regulated the ATM pathway to repress the autophagy response and promote the intracellular survival of *MTB*. The expression of exosomal miR-25–3p was also significantly increased in BCG-infected macrophages (64). By inhibiting the expression of DUSP10, mmu-miR-25–3p promoted the phosphorylation of ERK1/2 and thus enhanced the macrophage autophagy induced by BCG. This effectively suppressed the survival of intracellular *MTB* and promoted its elimination (**Figure 3**).

## 5 Exosomes as diagnostic biomarkers for TB

Commonly used clinical diagnostic methods, including sputum smear microscopy, serological testing, Xpert *MTB*/RIF testing, and quantitative polymerase chain reaction (qPCR) genetic testing, have been used for analyzing and evaluating TB incidence, development, and disease progression (65). The specificity, characterization, and simplicity of these methods are still not very satisfactory for diagnosing TB in the clinical setting. Novel and specific genetic and protein biomarkers have brought the possibility of early diagnosis and treatment monitoring for multiple disease (66–68). Mining nucleic acid and protein information in exosomes based on histologic techniques can provide comprehensive information about TB and enable more accurate prediction of TB prognosis. Since exosomes are lipid bilayer structures that are autocrine, they are more stable compared to freely circulating biomarkers and are effective at preventing the degradation of uploaded substances (7). At various phases of *MTB* infection, RNAs and protein cargoes are selectively encapsulated in exosomes, contributing to the development of potential targets for the diagnosis, prevention, and therapeutic monitoring of *MTB* (69).

### 5.1 Nucleic acids in exosomes

BCG infection of receptor immune cells, such as macrophages and DCs, contributes to the alteration and release of exosomal miRNAs by integrating the transcriptome. Mortaz et al. showed that the infection of MDMs with BCG facilitated the production of immune-associated miRNAs, including members of the Let-7 family, miR-155, miR-146a, miR-145, and miR-21, in exosomes (70). Zhan et al. confirmed that the levels of exosome components in macrophages were altered by BCG infection (71). After BCG infection, 20 exosomal miRNAs, including mmu-miR-27b-3p, mmu-miR-93–5p, mmu-miR-25–3p, mmu-miR-1198–5p, mmu-let-7c-5p and let-7a-5p, which were involved in multiple biological processes, were significantly up-regulated, and 7 exosomal miRNAs were down-regulated. These studies revealed that BCG alters the miRNA profile of exosomes in macrophages, but its significance in the fluid exosomes of TB patients and its potential as a diagnostic need further confirmation.

Exosomal RNAs are delivered to macrophages, affect transcription in recipient cells, stimulate the generation of inflammatory mediators, and trigger apoptosis. Exosomes released from *MTB*-infected macrophages harbor *MTB* proteins and lipids, and their miRNA abundance is lower than that in uninfected macrophages (72). Hu et al. found. that miR-20a, miR-20b, miR-26a, miR-106a, miR-191, and miR-486, were differentially expressed in TB patients (73). Combining exosomal miRNA and electronic health records (EHRs) enabled more accurate TB diagnosis. Alipoor et al. demonstrated a significant increase in miR-484, miR-425, and miR-96 in the serum of PTB patients, as well as the value of their expression in exosomes for the diagnosis of active PTB disease (74). Through multiple cellular infection models and sample validation, Kaushik et al. showed that plasma exosomal miR-185–5p was a potential biomarker for TB diagnosis (75).

Patients cured of TB are at high risk for lung cancer. Guio et al. analyzed the expression of circulating miRNAs in the blood exosomes of Peruvian patients with LTB, ATB, or lung adenocarcinoma, revealing that 24 miRNAs were dysregulated in these diseases (76). Lyu et al. reported that hsa-let-7e-5p, hsa-let-7d-5p, hsa-miR-450a-5p, and hsa-miR-140–5p, were specifically expressed in LTB, as were hsa-miR-1246, hsa-miR-2110, hsa-miR-370–3P, hsa-miR-28–3p, and hsa-miR-193b-5p in a TB cohort (77). They also identified several miRNA expression patterns that could be utilized to differentiate between LTB and ATB. Lv et al. examined exosomes extracted from clinical specimens of healthy controls, ATB, and LTB patients (69). Exosomes from LTB and ATB patients had numerous differentially expressed genes. For example, 12 and 14 genes from ATB were shown to be enriched in the lipid metabolism and extracellular matrix organization GO categories, respectively. These findings were consistent with the fact that *MTB* could utilize lipids from host cells for survival and the formation of granulomas. These findings suggested that RNAs could serve as potential targets for the development of diagnostic, preventive, and therapeutic strategies for TB.

Spinal TB is a common manifestation of extrapulmonary and osteoarticular TB, with severe neurological deficits and paraplegia. Early diagnosis of spinal TB is extremely important for controlling the progression of the disease, shortening the course of treatment, and preventing severe spinal deformities (78). Sun et al. found that 28 miRNAs were up-regulated and 34 miRNAs were down-regulated in patients with spinal TB. Among these miRNAs, miRNA-125b-5p was notably up-regulated and closely associated with the MAPK, TNF, Ras, Rap1, and PI3K-Akt pathways, demonstrating its potential as a diagnostic biomarker for spinal TB.

Long non-coding RNAs (lncRNAs) are classified as a type of RNA greater than 200 nucleotides in length that encode proteins of limited capacity but are involved in the regulation of transcription, mRNA processing, and post-transcriptional control. Dysregulation of lncRNAs in exosomes is closely associated with the clinical manifestations and diagnostic prognosis of multiple diseases, including inflammatory, neoplastic, and infectious diseases (79). Using a GEO dataset and PCR analysis, Fang et al. successfully identified 9 dysregulated lncRNAs (80). NONHSAT101518.2, NONHSAT067134.2, NONHSAT148822.1, and NONHSAT078957.2 were significantly down-regulated in the plasma of patients with ATB and were used to differentiate between ATB patients and healthy individuals, accompanied by high specificity and sensitivity.

### 5.2 Proteins in exosomes

*MTB* can survive in infected cells, and its secreted proteins can be integrated and encapsulated into host exosomes and released into the circulation. Exosomes are easily purified and stabilized, allowing for the enrichment of *MTB* analytes from complex serum protein mixtures (81). Infection with *MTB* leads to significant changes in the protein composition of exosomes from recipient cells (82). Proteomics and related methods based on proteomics have the potential to identify unexplored proteins and their functions and to facilitate TB diagnostics and vaccine development (83).

The expression of the CFL1 protein was significantly increased in macrophages infected with *Mycobacterium avium*, the supernatants of infected macrophages, and macrophage-derived exosomes, as well as in TB (6). These findings indicated the potential of CFL1 as a biomarker of *MTB* infection. Wang et al. investigated plasma exosomes from patients with rapidly growing NTM M. abscessus (MAB), slowly growing NTM M. intracellulare (MAC), and Mycobacterium tuberculosis (MTB) (84). The authors found that the expression of 18 proteins was markedly up-regulated in the plasma exosomes of MAB patients, while the expression of 6 and 10 proteins was up-regulated in MAC and *MTB* patients, respectively. MAB infection was associated with the HIF-1 signaling pathway and phagocytosis, while *MTB* infection affected the p53 signaling pathway. Huang et al. demonstrated that the Hsp16.3 protein was efficiently loaded in the exosome for transportation and was highly expressed in the exosomes of *MTB*-infected U937 cells (85). In addition, the level of the Hsp16.3 protein was significantly elevated in the blood exosomes of TB patients, which might serve as a diagnostic marker for TB.

Different stages of TB, such as ATB and LTB, lead to different degrees of host cell damage and viral genetic alterations, resulting in variations in exosome composition (86). These variations in the protein components of exosomes are expected to be important biomarkers for ATB and LTB. In a randomized controlled trial (RCT), Du et al. reported that down-regulated plasma exosomal S100A9/C4BPA was associated with a positive host response to LTB treatment (87). Kruh-Garcia et al. separated exosomes from serum samples of ATB patients and identified 20 characteristic proteins, including peptides of proteins such as 85B, antigen 85C, Apa, BfrB, GlcB, HspX, KatG, and Mpt64 (88). These proteins were unique markers of persistent ATB and LTB and were able to uncover a complicated biomarkers of TB states. Zhang et al. characterized the exosomal proteome in the sera of ATB patients and showed a significant increase in lipopolysaccharide-binding protein (LBP) but a significant decrease in CD36 and MHC-I in ATB exosomes (89). In addition, CD36 was downregulated in the serum of ATB patients and up-regulated in PBMCs and *MTB* H37Ra-infected macrophages.

## 6 Therapeutic applications of exosomes in TB treatment

### 6.1 Exosomes as vaccines

BCG is the only TB vaccine currently licensed for clinical use, but its efficacy in preventing TB infection and controlling TB transmission is limited, especially in adults. Therefore, the development of better efficacious TB vaccines is urgent. Exosomes isolated from BCG-infected macrophages can activate CD4+ and CD8+ T cells *in vitro* and induce naïve T-cell activation *in vivo*, indicating that the immunogenicity of exosomes is supportive of their use as vaccine candidates for anti-TB therapy. Through screening exosomal proteins derived from *MTB* or *MTB*-infected macrophages, Sharma et al. selected seven proteins, including DnaK, GrpE, LpqH, HBHA, LprA, LprG, and MPT83, with promising immunogenicity to construct a multi-epitope peptide vaccine, along with the TLR4 agonist, RpfE, as the vaccine adjuvant (90). They optimized the designed vaccine using computer modeling and simulation, as well as verified the vaccine efficacy through immunostimulation, demonstrating that it could be a prime candidate for the replacement of BCG.

Giri et al. discovered that BCG-infected macrophage exosomes contained MHC-I and MHC-II as well as stimulatory molecules that elicited the production of CD4+ T and CD8+ T cells (91). The obtained T cells were capable of secreting IFN-γ under BCG activation. Therefore, BCG-infected macrophage exosomes could be regarded as potent vaccine-like components that activate anti-*MTB* cellular immune responses. Furthermore, they confirmed that macrophages conditioned with *MTB* culture filtrate proteins (CFPs) released exosomes containing a variety of *MTB* proteins (92). These exosomal proteins were highly immunogenic and thus activate macrophages, DCs, and naïve T cells *in vivo*. In 2013, Cheng et al. showed that exosomes from CFP-treated macrophages induced a protective Th1-like immune response after inoculation of mice and were able to potentiate BCG immunization (93). Thus, this evidence suggested that exosomes composed of *MTB* antigens were potent inoculants for TB vaccines. Pei et al. detected the activity of vitamin C (VC) against BCG or H37Rv in macrophages (94). VC was effective in killing *MTB*, which was dependent on reactive oxygen species (ROS) formation and activation of the oxidative stress pathway, and VC-treated RAW 264.7 exosomes were able to kill BCG *in vitro*, thus confirming the potential of VC and VC-pretreated macrophage exosomes for the treatment of TB.

### 6.2 Using MSC exosomes as carriers

**Figure 4:**
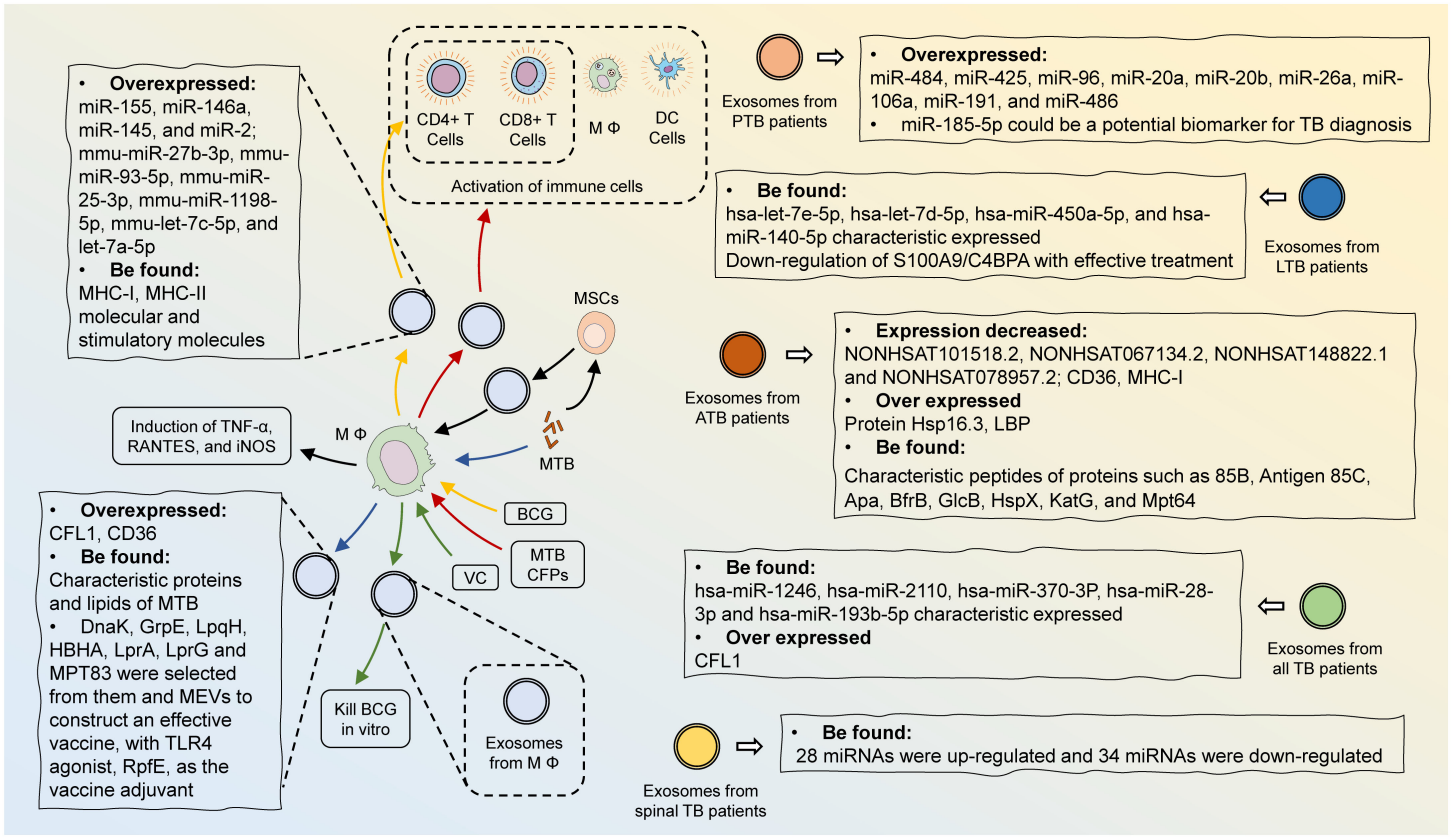
The potential of exosomes in the diagnosis and treatment of TB. Characteristic proteins and lipids of *MTB*, along with high levels of CFL1 and CD36, are found in exosomes from MTB-infected macrophages. MSCs infected with *MTB* secrete exosomes to induce the production of TNF-α, RANTES, and iNOS. The exosomes released from BCG-stimulated macrophages contain large amounts of miR-155, miR-146a, miR-145, miR-2, mmu-miR-27b-3p, mmu-miR-93–5p, mmu-miR-25–3p, mmu-miR-1198–5p, mmu-let-7c-5p, and let-7a-5p. MHC-I, MHC-II, and stimulatory molecules are also present and contribute to the activation of CD4+ T cells and CD8+ T cells. Exosomes containing multiple *MTB* proteins can be released from macrophages treated with *MTB* CFPs, resulting in the activation of numerous immune cells.

Mesenchymal stromal cells (MSCs) are a class of multidirectionally differentiated, paracrine-rich stem cell types that secrete exosomes of the same composition and abundance as the parent cells (95). Compared to MSCs, exosomes are more stable for the treatment of clinical diseases and have a lower likelihood of immune rejection after *in vivo* allogeneic administration, non-teratogenicity, low-temperature resistance, and easy transportation and preservation, providing alternative therapies for TB treatment. Liu et al. noted that exosomes derived from *MTB*-infected MSCs (Exo-MSCs-*MTB*) could be internalized by macrophages and thus induced TNF-α, RANTES, and iNOS to promote inflammatory response (96). Exo-MSCs-*MTB* were injected into mice and triggered a strong inflammatory response. This suggested that Exo-MSCs-*MTB* might be a potentially effective therapeutic strategy for TB. Central nervous system TB (CNS-TB) is the most devastating form of extrapulmonary TB with a high mortality rate and includes tuberculous meningitis, intracranial tuberculous tumors, and tuberculous arachnoiditis. Li et al. designed a novel BMSC-exosome-based nanoparticle, ANG-Exo-RIF, loaded with rifampicin and the brain-targeting peptide angiopep-2 (97). ANG-Exo-RIF exhibited high targeting ability and penetration, excellent anti-TB activity, and good biocompatibility, which is promising for CNS-TB treatment (**Figure 4**).

## 7 Limitations and perspectives

Exosomes are important mediators of host-pathogen interactions during *MTB* infection, providing a new perspective on TB development. Exosomes contain a variety of biologically active molecules, which not only participate in the development of TB, but also can serve as stable biomarkers for fluid diagnosis. At the same time, exosomes are also endowed with capabilities for treating TB. Currently, there are still some significant challenges and concerns that deserve to be addressed in practice, including exosome obtainment, the mechanism of exosomes in TB, and exosomes in TB diagnosis and treatment.

First, the acquisition and purification of exosomes in TB is a limiting factor that needs to be considered. The process of exosome isolation is highly susceptible to contamination. Due to the highly heterogeneous nature of exosomes and nonspecific surface labeling, isolating different types of exosomes and distinguishing their different sources can be difficult. Currently, commonly used methods for exosome isolation include size exclusion, affinity-based chromatography, and ultracentrifugation (98). However, each technology has certain advantages and disadvantages. Inconsistent methods of isolation and extraction of TB exosomes by different research groups may lead to differences and bias in research conclusions. Therefore, how to better extract and isolate exosomes and control their quality standards is an urgent challenge.

Second, existing mechanistic studies on the exosomes of TB are not sophisticated enough. Exosome-mediated interactions between *MTB* and the host may be far more complex than previously believed. Exosomes are the carriers for *MTB* survival in host cells, and at the same time, exosomes have good immunogenicity and can activate the immune system, which in turn removes *MTB*. Exosomes derived from *MTB* may coalesce with host cell-derived exosomes, both in abundance and compositional attributes, thereby collectively modulating the biological state of the infected cells. In addition, the specific effects of exosome levels, sources, and composition on the development of TB disease in patients with different infection statuses and stages, such as ATB and LTB patients, have not been clearly described. Since both MTB-infected cells and immune cells are capable of secreting exosomes and can influence their functions, it is not known which cellular exosomes and components are dominant.

Third, the diagnostic efficacy of exosomes in TB needs to be exploited. Multi-omics based cell and sample data mining has yielded novel markers and offers vast prospects for disease diagnosis (99, 100). The protective effect of exosomes allows ncRNAs to be well stabilized in exosomes, which provides a basis for their development as diagnostic markers. Several miRNA combinations have been proposed to have potential as TB diagnostic markers, however, other nucleic acid species, such as lncRNAs and circRNAs, have rarely been investigated for TB diagnosis and deserve to be identified by large-scale gene sequencing. Uniform evaluation of models for single-gene diagnostics or multigene constructs is difficult because most studies have inconsistent sample sizes, isolation methods, and assays. It is important to emphasize that all of these biomarkers can currently only serve as useful supplements for the routine clinical diagnosis of TB, not as replacements. In addition, the problems of cost and accuracy of exosome isolation remain to be surmounted, and the establishment of a standardized procedure for exosome RNA analysis with sufficiently convincing results is a direction for future research.

Finally, all relevant therapeutic strategies and targets should ultimately be available for clinical use in order to be meaningful (101–103). the use of exosomes for the development of TB vaccines has both potential and risks and is still in the theoretical research phase. Exosomes can be used as targets for TB treatment and as carriers for drug piggyback therapies, opening up a wide range of possibilities for targeted TB therapies. TB-derived exosomes are immunogenic and can activate both humoral and cellular immunity. Genetic engineering editing or drug pretreatment based on TB exosomes or TB-infected macrophage exosomes may enhance the immunocidal potency against TB and improve the stability of exosomes and the safety of therapy.

## 8 Conclusions

Exosomes are mediators of information transfer and interactions between TB and host cells, and play an indispensable role in the development of TB by regulating immune defenses, inflammatory responses, metabolic pathways, and modulating the mode of cell death. In addition, exosomes and components such as proteins and ncRNAs encapsulated in exosomes are characterized by spatial and temporal alterations, thus can be effective targets for TB diagnosis and treatment. Continued basic and clinical research on exosomes will provide a powerful strategy for the treatment of TB.

## Author contributions

XS: Writing – original draft, Writing – review & editing. WL: Writing – original draft, Writing – review & editing. LZ: Writing – original draft, Writing – review & editing. KF: Writing – original draft, Writing – review & editing. FQ: Writing – original draft, Writing – review & editing. LS: Writing – original draft, Writing – review & editing. FG: Writing – original draft, Writing – review & editing. CZ: Writing – original draft, Writing – review & editing.
